# Analysis of the Salivary Microbiome in Obstructive Sleep Apnea Syndrome Patients

**DOI:** 10.1155/2020/6682020

**Published:** 2020-12-23

**Authors:** Peizeng Jia, Jianyin Zou, Shankai Yin, Feng Chen, Hongliang Yi, Qian Zhang

**Affiliations:** ^1^Department of Orthodontics, Peking University School and Hospital of Stomatology, Beijing, China; ^2^Department of Otolaryngology Head and Neck Surgery and Center of Sleep Medicine, Shanghai Jiao Tong University Affiliated Sixth People's Hospital, Shanghai, China; ^3^Central Laboratory, Peking University School and Hospital of Stomatology, Beijing, China

## Abstract

**Background:**

Oral microbiota plays an important role in oral and systemic diseases, while few reports referred to obstructive sleep apnea syndrome (OSAS). Thus, this study aimed to explore the different salivary microbiome in patients with OSAS and controls.

**Materials and Methods:**

Saliva was collected from 15 OSAS patients and nine healthy controls, and bacterial genomic DNA was extracted for 16S rRNA amplicon sequencing based on the Illumina platform.

**Results:**

The alpha and beta diversities were not significantly different between patients with OSAS and controls. The main phyla in the two groups were Firmicutes, Actinobacteria, Bacteroidetes, Proteobacteria, and Fusobacteria, which accounted for 95% of the abundance. The main genera were *Streptococcus*, *Rothia*, *Actinomyces*, *Prevotella*, and *Neisseria.* Based on the genus and operational taxonomic units, *Peptostreptococcus*, *Alloprevotella*, and *Granulicatella* were enriched in controls, while only *Scardovia* species were significantly more abundant in patients with OSAS.

**Conclusions:**

There was no significant difference in the relative abundance of bacteria between OSAS and controls. So, further studies will need to focus on the metagenome of bacteria in OSAS patients.

## 1. Introduction

Obstructive sleep apnea syndrome is a sleep-breath disorder caused by upper airway obstruction and characterized by recurrent hypoxemia and daytime sleepiness [1, 2]. It can occur at any age and lead to poor quality of life [3]. Moreover, the prevalence and burden of OSAS are expected to increase in the future. The underlying pathology of OSAS may be intermittent hypoxemia and hypercapnia of the upper airway, leading to local and systemic inflammatory responses [4, 5].

The etiology of OSAS is not understood clearly. Some studies have shown that there is a close relationship between microorganisms and OSAS. Intermittent hypoxia regulates hypoxia/reoxygenation cycles in the upper airway and gut, which increases the bacterial diversity in OSAS patients. Based on this, microbial changes can affect the inflammatory process in different mucosal tissues, including nasal fluid [6], tonsil [7], and gut [8]. The relative abundances of *Streptococcus*, *Prevotella*, and *Veillonella* were higher in the nasal fluid of severe OSAS patients than those in healthy subjects. *Streptococcus salivarius*, *Prevotella* spp., and *Terrahaemophilus aromaticivorans* were more common on adenoids from patients with OSAS [7]. Additionally, the altered microbiome in severe OSAS patients was associated with inflammatory biomarkers (e.g., inflammatory cells, interleukin IL-8, and IL-6) [6]. Also, proinflammatory cytokines were elevated in the saliva and serum of OSAS patients [9]. All this proved that microbial-host interaction might play an important role in immune response in OSAS patients.

Bacteria, which have colonized every location of the human body, are linked to human health. The oral cavity is an important exchange medium between exogenous substances and the internal environment [10]. Many studies have focused on the link between oral microbiome and systemic disease [11], for example, chronic kidney disease [12], diabetes [13], and obesity [14], because OSAS can also cause some complications such as hypertension and other cardiovascular diseases [15]. Thus, specific bacterial microenvironments may be present in the oral cavity of OSAS patients. However, there are only a few studies concerning this issue [16, 17]. The two studies demonstrated that OSAS is closely linked with periodontitis and the buccal mucosal microbiome was significantly altered in pediatric OSAS patients, respectively.

Saliva, as a representative sample of the oral ecosystem has become an important sample matrix in bioanalytics and reflects systemic conditions [18]. Salivary microbiota is associated with oral and systemic diseases and mediated inflammatory responses. Therefore, we aimed to explore the bacterial composition and community structure of saliva in patients with severe OSAS and controls with 16S rRNA high-throughput sequencing to determine the relation between OSAS and oral microbiome.

## 2. Materials and Methods

### 2.1. Subjects

The study included unrelated subjects suspected of having OSAS who were admitted to the Sleep Center of the Affiliated Sixth People's Hospital, Shanghai Jiao Tong University meanwhile. Ten subjects who did not snore were also recruited to complete the overnight polysomnography test. The Institutional Ethics Committee of the Hospital of Shanghai Jiao Tong University approved the study (protocol reference number 2018-KY-013 (K)). Informed consent was obtained from all participants. All participants were asked to complete a uniform questionnaire containing questions regarding current and previous illnesses and medical treatments. Subjects with the following indexes were excluded: systemic diseases (e.g., hypertension and diabetes), oral disease (e.g., periodontitis and dental caries), smoking, antibiotics applied for less than three months, and any treatment with adenoids.

The diagnostic criterion of OSAS patients was the apnea-hypopnea index (AHI; mean number of apneas or hypopneas per hour), with apnea defined as the cessation of flow for > 10 s and hypopnea as *a* > 50% reduction inflow. An AHI of 5–15 is considered mild, 16–30 is moderate, and >30 is considered severe. Finally, only severe OSAS patients and healthy subjects were included. The diagnostic evaluator has a rich clinical experience for eight years and has been employed as a Technical Section Member of the Chinese Medical Doctors Association Sleep Medicine Specialized Committee.

### 2.2. Saliva Collection

After the overnight polysomnography test, all subjects were required not to drink and eat for two hours and clean their mouth of obvious food residue on the oral mucosa and teeth before sampling. Unstimulated saliva (2 ml) was collected in a 50 ml sterile tube, placed on ice, delivered to the laboratory. Each sample was centrifuged at 8000 rpm for 10 min at 4°C, and the precipitate was collected and stored at −80°C for DNA extraction.

### 2.3. DNA Extraction and Sequencing

The 24 saliva precipitates were digested by lysozyme (20 mg/ml) and digested at 37°C for 30 min, and then bacterial genomic DNA was extracted using a QIAamp DNA Mini Kit (Qiagen, Hilden, Germany) according to the instructions. The DNA quality was determined by the OD_260/280_ ratio (1.8–2.0), using a Nanodrop 8000 spectrophotometer (Thermo Fisher Scientific, USA). DNA integrity was verified by 1% agarose gel electrophoresis. High-quality DNA was stored at −20°C for further sequencing.

An equal DNA concentration (10 ng/*μ*l) from 24 samples was used for 16S rRNA gene amplification of V3–V4 regions (primers: F341 : ACTCCTACGGGRSGCAGCAG, R806 : GGACTACVVGGGTATCTAATC) [19] with an 8 bp unique index inserted at the 5′ end to distinguish the different samples. PCR was performed using a KAPA HiFi HotStart ReadyMix PCR Kit; the products were purified using an AxyPrep DNA Gel Extraction Kit (Axygen, USA). The extracted products were quantified with Qubit 3.0 and real-time PCR to conduct libraries following the instructions according to the manufacturer's instructions. The libraries were sequenced using the Illumina HiSeq PE250 platform by Shanghai Realbio Technology Co., Ltd. (Shanghai, China). The raw sequence data have been submitted to the NCBI with the accession number PRJNA544600.

### 2.4. Data Processing

The raw sequence data were assigned to each sample using the unique barcode sequence. The paired-end reads were assembled using Pandaseq software [20] based on the overlap region. High-quality reads were selected using Usearch software (version 7.0190) based on the following criteria: an average value was >20, a base number containing *N* < 3, and an average length was 220–500. After singletons were filtered, the clean reads were clustered into operational taxonomic units (OTUs) by 97% similarity using UPARSE [21], and chimeras were removed using Usearch. The sequence with the highest abundance was selected from each OTU and used as the representative sequence of the OTU. The representative sequence was classified against the Ribosomal Database Project (RDP) database and Human Oral Microbiome Database to assign microbial taxa (phylum, class, order, family, and genus) for each sample.

All subsequent analyses were conducted using QIIME (version 1.9.1) [22]. The OTUs of 24 samples were used for further analysis. To identify the alpha diversity and beta diversity, the same number of clean sequences was chosen from each sample to reduce the sequence depth factor. Principal coordinates analysis (PCoA) was performed to analyze the structure of microbial communities in controls and patients with OSAS based on the OTU level.

### 2.5. Statistical Analysis

Clinical data (age, body mass index (BMI), and apnea-hypopnea index (AHI)) were compared by independent-samples *t*-test. The index of sex was compared by the chi-square test, and the other indexes of mean SaO_2_, minimum SaO_2_, and oxygen desaturation index (ODI) were compared by the Wilcoxon rank-sum test, respectively, using SPSS software (version 19.0). The alpha diversity (Chao1, Observed OTUs, PD whole tree, Shannon) and bacterial composition were tested using the Wilcoxon rank-sum test. LEfse analysis was based on Wilcoxon rank-sum test and with the threshold of logarithmic linear discriminant analysis (LDA) score set to 2.0. The PCoA of beta diversity was performed by the ANOSIM test. *P* value < 0.05 was considered statistically significant.

## 3. Results

### 3.1. Basic Information

Nine controls and 15 patients with severe OSAS patients were chosen for this study. There were no significant differences in sex, age, and body mass index (BMI) between the two groups (Table 1). The AHI and ODI indexes in OSAS patients were significantly higher than those in the controls. Because OSAS patients have apnea at night, which will lead to hypoxia, so the mean SaO_2_ and minimum SaO_2_ indexes were significantly lower in OSAS patients than those in the controls.

Sequencing yielded 862,398 clean reads after quality assessment and filtering, with an average of 35,933 reads per sample. The number of OTUs per sample was 152–251.

### 3.2. Biodiversity of the Salivary Microbiome

**Figure 1:**
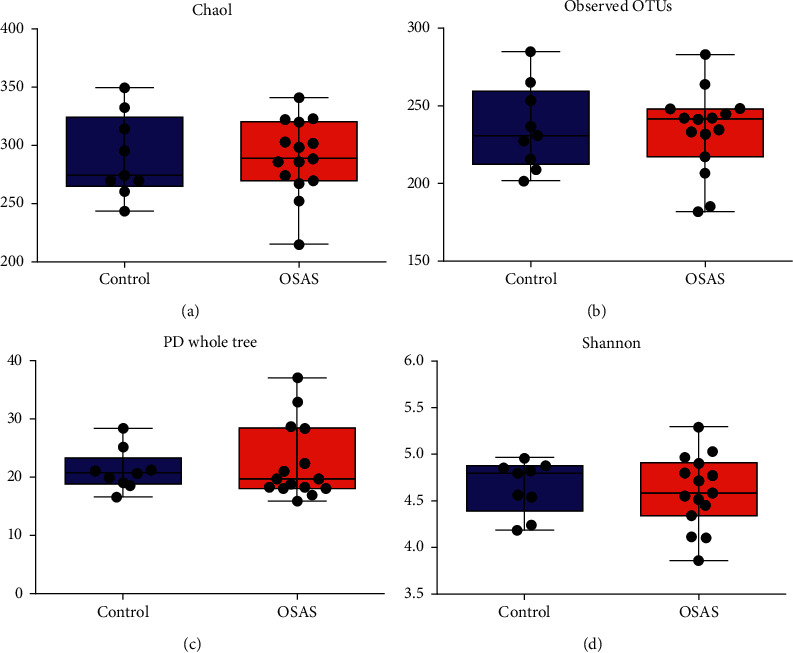
Comparison of salivary microbiome alpha diversity in patients with severe obstructive sleep apnea syndrome (OSAS) and controls. (a, b) Community richness (chao1 and observed operational taxonomic units), (c) comparison of the bacterial evolutionary distance, and (d) the Shannon index. All of the indexes were tested using the Wilcoxon rank-sum test. The significant difference was set by *P* < 0.05. Box and whisker plots were indicated medium, minimum, and maximum values. All the samples were shown in the plot.

We conducted alpha and beta diversity analyses to explore the microbial community composition and structure in OSAS patients and controls. The observed OTUs and the Chao index, which represent the microbial richness, and the Shannon index were no significant differences between OSAS patients and controls (Figure 1).

**Figure 2:**
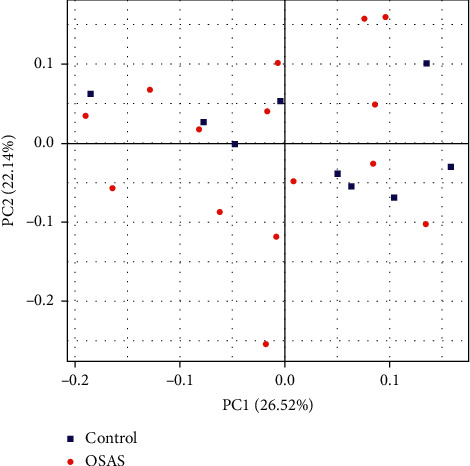
PCoA analysis of the salivary microbiome of patients with severe OSAS and controls using principal coordinate analysis. The analysis was based on UniFrac distance. Significant differences were assessed using Anosim analysis. Significance was indicated by *P* < 0.05.

Beta diversity is used to describe phylogenetic differences in microbial communities between diseased and controls. This method can present the bacterial difference between two groups based on the distance. As shown in Figure 2, the OSAS and healthy samples overlapped, and there was no apparent difference in distribution between the two groups, with the principal components of 26.52% and 22.14%. ANOSIM analysis also showed that there was no significant difference in bacterial composition and structure between the controls and patients with OSAS (*P* > 0.05) (Figure 2). PCoA analysis was conducted using weighted UniFrac distances based on the OTU level, which showed that there was a similar bacterial environment between controls and patients with OSAS.

### 3.3. Bacterial Composition of Saliva in OSAS Patients and Controls

**Figure 3:**
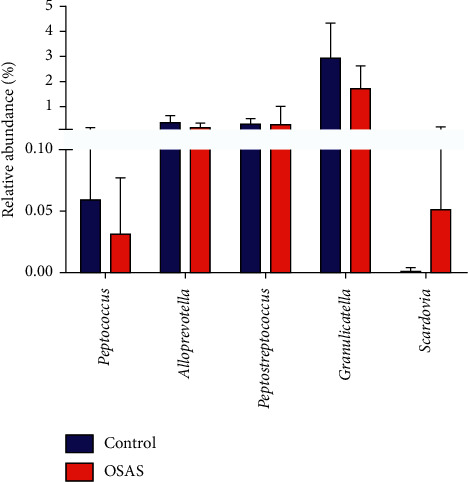
Significantly different genera in patients with severe OSAS and controls. The significantly different genera (prevalence > 50% in all samples in each group) between the two groups were accessed using the Wilcoxon test based on the relative abundance values. The significant difference was set by *P* < 0.05. The error bars were presented as mean.

We analyzed the relative abundance of microbial taxa at the phylum, class, order, family, and genus levels. The main phyla in the two groups were Firmicutes, Actinobacteria, Bacteroidetes, Proteobacteria, and Fusobacteria, which accounted for 95% of the abundance. At the genus level, *Streptococcus*, *Rothia*, *Actinomyces*, *Prevotella,* and *Neisseria* were the most abundant in the two groups, and there were no significant differences in these genera between the two groups (Figure 1). The proportions of *Peptococcus*, *Peptostreptococcus*, *Alloprevotella*, and *Granulicatella* were less abundant in patients with OSAS compared with controls; only the genus *Scardovia* had a significantly higher abundance in patients with OSAS (Figure 3).

**Figure 4:**
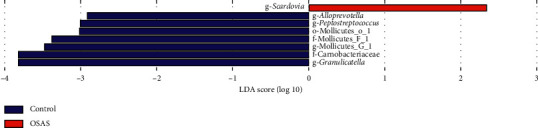
Taxonomic differences in salivary samples from patients with severe OSAS and controls. The enriched bacteria between the two groups based on linear discriminant analysis effect size analysis (LEFse). LDA > 2 is labeled.

The linear discriminant analysis effect size (LEfse) method was used to analyze the influence of bacteria on health and disease, with LDA > 2 labeled based on the OTU level, which considered the statistical significance and biological correlation. This method revealed the influence of significantly different bacteria on the two groups. Genera such as *Peptostreptococcus*, *Alloprevotella*, and *Granulicatella* were enriched in healthy controls, while *Scardovia* was significantly more abundant in patients with OSAS (Figure 4).

## 4. Discussion

Our study revealed the bacterial composition and diversity of saliva microbiome in adult OSAS, demonstrating that the bacterial microenvironment of saliva was relatively stable compared with controls. No significant difference in the alpha diversity of the salivary microbiome was indicated between patients with severe OSAS and controls in our results. Moreover, genera such as *Peptostreptococcus*, *Alloprevotella*, and *Granulicatella* were enriched in healthy controls while *Scardovia* was enriched in patients with OSAS. We know that the oral cavity does not belong to the upper airway system, so the saliva environments of patients with OSAS and controls may be similar.

OSAS is a significant risk factor for hypertension, cardiovascular disease, and metabolic disorders such as obesity [14] and diabetes [13]. Bacteria play an important role in these processes. It is possible that OSAS leads to gut hypoxia and hypercapnia and increased sympathetic activity, which results in gut dysbiosis [23, 24]. A study has showed higher relative abundances of *Porphyromonas* and *Aggregatibacter* and elevated proinflammatory cytokines in patients with OSAHS compared with controls without OSAHS [25]. Recent studies have also shown that due to the reflux of oropharyngeal oral secretions during sleep, oral bacteria were detected in the nasal lavage of patients with OSAS [6]. The oral environment represents an interface between the internal and external environments, and it is easily influenced by the internal environment. The metabolic products of oral bacteria can be recycled into the blood circulation and are involved in local and systemic immune responses, which may accelerate the progression of systemic diseases [26, 27]. It has also been shown that patients with OSAS experience local (upper airway) and peripheral (systemic) inflammation. Thus, maintaining a relatively good oral environment is important for patients with OSAS.

Studies found that the composition and the metabolomics profile of the oral microbiome were significantly altered in pediatric OSAS [17]. Although there were no significant differences in the overall phylogenetic structure of the salivary microbiome, several bacteria were also altered because of the oxygen environment. *Peptostreptococcus*, *Alloprevotella*, and *Granulicatella* were enriched in controls, while *Scardovia* was enriched in OSAS patients. *Peptostreptococcus* is the most common Gram-positive anaerobe found in the oral cavity of healthy and patients. Many diseases are caused by this bacterium, including endocarditis [28] and root canal infections [29]. *Alloprevotella* is anaerobic Gram-negative rods isolated from the normal oral and intestinal bacterial population. Although not considered pathogenic, the strains were saccharolytic ability and produced acetic and succinic acids in the oral cavity [30]. All of the above identified genera were common in the samples obtained from controls. Some species in the genus *Scardovia* were found to be related to dental caries [31], which indicated that OSAS patients might tend to have oral disease. The oral condition change was caused by a combination of bacteria rather than by a single bacterium. Although there was no difference in the salivary microbiome of the controls compared with OSAS patients, OSAS patients should also pay attention to oral health, because they can be easily infected.

There are some limitations to the study. First, the sample size was relatively small to reach our conclusion. Thus, a larger validation study and metagenomic analysis would be useful to support our results. Second, a dietary diary should be considered to reduce the influence of food on the oral salivary microbiome. Finally, different oral microbiota was present at different oral sites; therefore, saliva collection alone cannot represent the entire oral microbiome.

## 5. Conclusion

This study applied 16S rRNA gene sequencing technology to analyze the salivary microbiome between OSAS patients and controls. The results showed that there were no significant differences in the bacterial diversity and phylogenetic structure of the salivary microbiota. However, the relative abundances of *Peptococcus*, *Peptostreptococcus*, *Alloprevotella*, and *Granulicatella* were lower and only the genus *Scardovia* was enriched in the saliva samples from OSAS patients compared with controls.

## Figures and Tables

**Table 1:** The basic clinical information of OSAS patients and control subjects.

Characteristics	OSAS	Controls	*P* value
No. of subjects	15	9	—
Age (years)	47.0 ± 9.5^a^	40.2 ± 9.4^a^	0.103
Sex (male/female)	13/2	6/3	0.326
BMI^d^ (kg/m^2^)	27.0 ± 3.8^a^	28.5 ± 6.4^a^	0.453
AHI^c^ (events/h)	54.4 ± 19.0^a^	2.7 ± 1.2^a^	<0.001
Mean SaO_2_ (%)	93 (91–94)^b^	96 (95–97)^b^	0.007
Minimum SaO_2_ (%)	74 (67–81)^b^	93 (91–94)^b^	<0.001
ODI^e^ (events/h)	58.0 (47.0–69.1)^b^	3.4 (1.7–5.2)^b^	<0.001

^a^Values are presented as means ± SD. ^b^Values are presented as mean (with 95% confidence intervals) as appropriate. ^c^AHI = apnea-hypopnea index. ^d^BMI = body mass index. ^e^ODI = oxygen desaturation index. The age and BHI and AHI indexes were compared by independent-samples *t*-test, and the sex index was compared by the chi-square test; the other indexes of mean SaO_2_, minimum SaO_2_, and ODI indexes were compared by the Wilcoxon rank-sum test, respectively, using SPSS software.

## Data Availability

The data used to support the findings of the study are available from the corresponding author upon request, and the raw data had been uploaded to the NCBI with the accession number PRJNA544600.

## Supplementary Materials

Supplementary Materials SFigure 1: bacterial composition at the phylum and genus level. (A) Phylum level and (B) genus level.
