# Supplementary figures and images for: Analysis of the Salivary Microbiome in Obstructive Sleep Apnea Syndrome Patients
Source: Can J Infect Dis Med Microbiol. 2020 Dec 23;2020:6682020. doi: 10.1155/2020/6682020 (PMC7803107; doi:10.1155/2020/6682020)

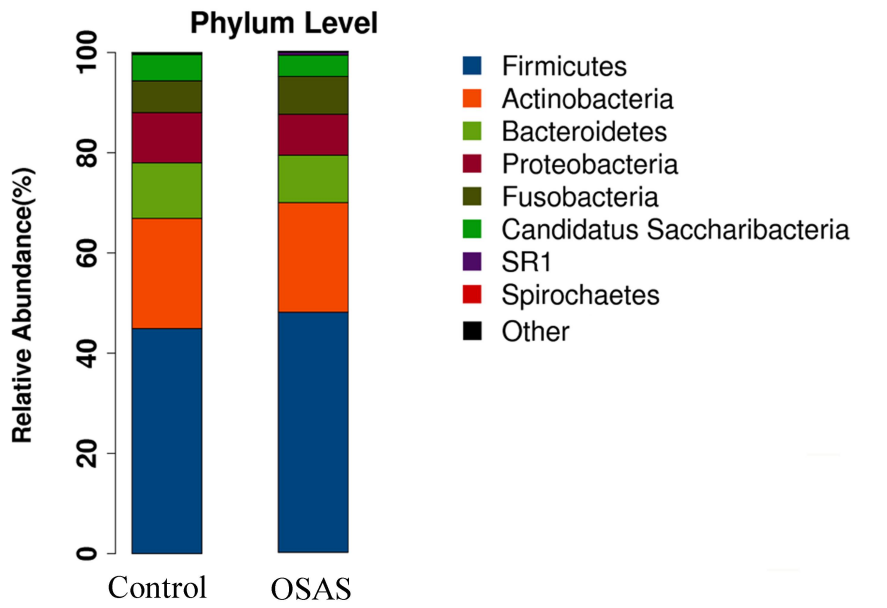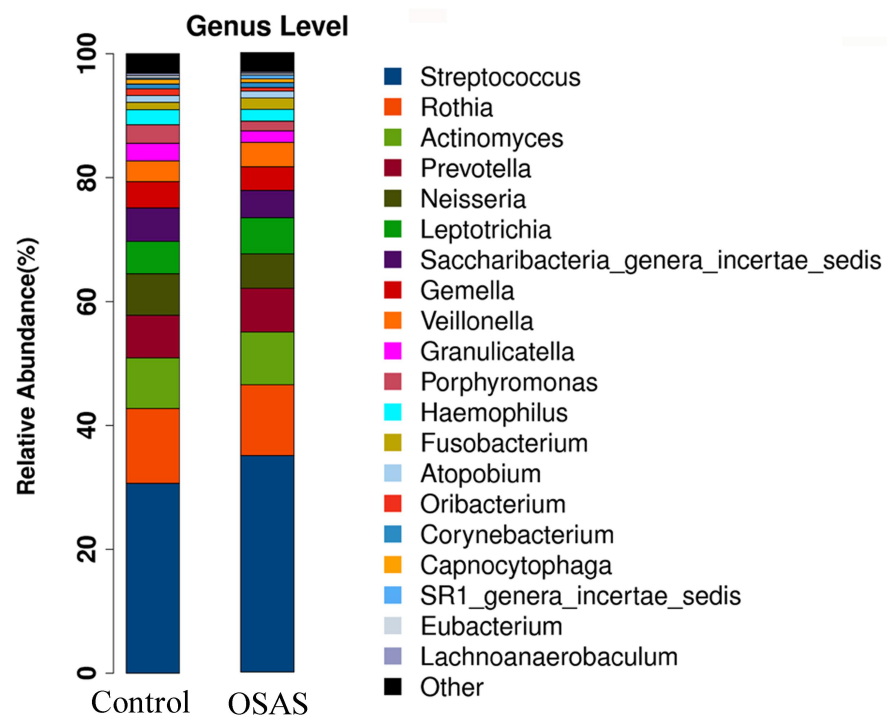

Supplement: Supplementary Materials — SFigure 1: bacterial composition at the phylum and genus level. (A) Phylum level and (B) genus level. [file 6682020.f1.pdf]
